# Development and evaluation of the measurement properties of a generic questionnaire measuring patient perceptions of person-centred care

**DOI:** 10.1186/s12913-020-05770-w

**Published:** 2020-10-20

**Authors:** Helena Fridberg, Lars Wallin, Catarina Wallengren, Anders Kottorp, Henrietta Forsman, Malin Tistad

**Affiliations:** 1 School of Education, Health and Social Studies, Dalarna University, Falun, Sweden; 2 Institute of Health and Care Sciences and University of Gothenburg Centre for Person-Centred Care, Sahlgrenska Academy at the University of Gothenburg, Gothenburg, Sweden; 3 Faculty of Health and Society, Malmö University, Malmö, Sweden; 4 Department of Neurobiology, Care Sciences and Society, Karolinska Institutet, Stockholm, Sweden

**Keywords:** Person-centred care, Rasch analysis, Mixed methods, Qualitative content analysis, Questionnaire development, Delphi study, Content validity index

## Abstract

**Background:**

Implementation of person-centred care (PCC) is a challenging undertaking. Thus, a call has been issued for a robust and generic instrument to measure and enable evaluation of PCC across settings and patient groups. This study aimed to develop a generic questionnaire measuring patients’ perceptions of PCC. Further aims were to evaluate its content and measurement properties using a mixed-methods approach entailing Rasch and qualitative content analyses.

**Methods:**

The study was conducted in three iterative phases. Phase one included six key informants to gain a broad view of the concept. Phase two entailed a Delphi study involving two rounds with eight experts who generated ratings on relevance, readability, comprehensiveness and suggestions for revision. Data were analysed using the Item Content Validity Index in conjunction with qualitative comments to improve the questionnaire. Phase three was performed using a mixed-methods design. Quantitative data were collected from patients (*n* = 553) responding to the questionnaire who were recruited from six in- and outpatient care units in a health care region in Sweden. Data was analysed using the Rasch measurement model. Qualitative data were based on the respondents’ free-text comments, cognitive interviews (*n* = 10) and field notes, and then analysed with deductive content analysis.

**Results:**

A questionnaire was developed and operationalised based on the information given by key informants in phase one and then validated for its content by experts in phase two. In phase three Rasch analyses revealed problems with targeting, thresholds and two misfitting items. These problems were corroborated by data from the qualitative analyses, which also revealed some issues of wording and interpretation of items. When thresholds were resolved and two items removed, the questionnaire met the assumptions of the Rasch model.

**Conclusions:**

Experts gave the questionnaire content high ratings and it met measurement requirements assumed by the Rasch model after revisions. Those problems on targeting that remain need to be addressed in future studies. Meanwhile, we regard the questionnaire as of sufficient quality to be useful in benchmarking PCC.

## Background

There is a worldwide effort to increase people’s empowerment, rights and patient participation in health care [1, 2]. Person-centred care (PCC) is a core phenomenon within this endeavour that is growing in popularity among policymakers, leaders, health care professionals (HCP) and other stakeholders in health care [1, 3]. PCC can be viewed as the co-creation of health care in the actual meeting between HCPs and patients. Moreover, the meaning of PCC sometimes includes work routines, support structures and care processes within and across organisations [1]. It can be regarded either as a goal in its own right with a focus on ethical factors such as a commitment to strive for the common good, viewing the patient as a person with an entire life beyond the medical perspective, or as a means through which other health care outcomes can be reached [1]. However, implementation of PCC is a challenging undertaking and a lack of consensus regarding how to define, conceptualise, practice and measure PCC is commonly reported [4–8].

Researchers at The Gothenburg University Centre for Person-centred Care (GPCC) conceptualise and define PCC largely from a philosophical foundation based on relationship ethics and person-centredness with a shift from the term patient to person [9–11]. GPCCs definition of PCC, with its starting point in the concept of person, implies that the patient is perceived as something more than their illness [9]. According to philosophical teachings by Ricoeur [11], a person can take responsibility for themselves and their life even though they are suffering from an illness. This makes the patient and the HCP mutually dependent on each other as they together contribute in the co-creation of the care [11]. The concept of person requires that the HCPs take ethical responsibility by paying attention to the person behind the patient and acknowledge the patient as an expert on their everyday life situation, goals and wishes for health care [12]. Recognising the person strengthens the patient as a partner in their own care process [12]. Researchers at GPCC have translated the ethical underpinnings of PCC into clinical practice through three routines aimed at reinforcing the partnership between the HCP and the patient [9, 12, 13]. The three core routines are: 1) initiating a partnership by listening to patients’ narratives to understand their resources, abilities and personal wishes for illness self-management; 2) working in partnership by discussing and co-creating medical investigations, care, treatment or rehabilitation plans together with patients; and 3) safeguarding the partnership where decisions and goals, shared between patients and health care professionals, are agreed upon, documented and signed. These routines should not be seen as separate but as interrelated and used in a non-linear manner that moves back and forth in the meeting between HCPs and patients [12]. GPCC’s ethical approach based on these core routines is also in accordance with the newly published European Standard on PCC [9, 12, 14].

Results from randomised controlled trials involving interventions thought to comply with PCC reveal positive patient outcomes, including a shortened hospital stay [15], lower patient anxiety and uncertainty [16], reduced agitation [17] and improved general self-efficacy [18]. However, PCC as viewed from the patient’s perspective is seldom measured and reported in those intervention studies. This creates a knowledge gap concerning to what extent the patients perceived the intervention to be in line with values purported by the ethical claims made by the researchers [5]. It is therefore difficult to deduce what active PCC actions and activities each intervention entailed, or the level of PCC delivered [1, 5, 19]. Even though there is a wide range of measures of patients’ perceptions of PCC these are often based on samples in a specific setting or disease group or related to the care given by a single provider [8, 20–22]. Another common approach in available PCC measures has been to target specific components within PCC [8], such as shared decision making [23], person-centred communication [24] and empathy [25]. A lack of a universally accepted definition of PCC along with an agreed generic outcome measure targeting PCC from a patient perspective prevents comparing and generalising studies [8, 19, 20, 26].

The movement towards increasing the presence of PCC is apparent in Sweden, where policymakers for some time have been pushing for the implementation of PCC as a means of improving the quality of health care [27]. As of 2018, more than half of the regions in Sweden have made an active decision to implement PCC, with the numbers continuing to increase [28]. While GPCC has been a significant player in PCC research in Sweden, many regions have adopted a conceptualisation of PCC in line with that of this centre.

Both internationally and across health care regions in Sweden several requirements have been identified to facilitate the implementation of PCC, one being the need to measure PCC to monitor change over time and having opportunities to make comparisons across units, regions and countries [1, 28]. There are a range of questionnaires aimed at measuring PCC targeted for specific contexts, patients’ groups and HCPs [8, 20, 22]. However, to our knowledge, there is a lack of robust [29], generic questionnaires which measure and compare patients’ perceptions of PCC in line with GPCCs conceptualisation across health care settings, patient groups, and health care professions. Thus, this study aimed to develop a generic questionnaire measuring patients’ perceptions of PCC and to evaluate its content validity and measurement properties.

## Methods

A collaboration with representatives of the National Patient Survey at The Swedish Association of Local Authorities and Regions (SALAR) was initiated from the outset in this development process to enhance the opportunities for broad implementation and use in real-world settings. SALAR runs The National Patient Survey, which is a generic questionnaire capturing patients’ perceptions of their health care in inpatient and outpatient settings [30]. This survey has been developed in cooperation with patients and is conceptualised in six separate dimensions, including a basic item pool with 22 generic items aimed at comparisons across diverse care specialties and another 10 items mainly intended for primary care settings. The dimensions are *emotional support, information and knowledge, involvement and participation, continuity and coordination, accessibility, respect and approach, and general impression* [30]. The questionnaire aims at supporting health care improvements from a patient perspective, evaluating care between health care units and functioning as a control and management tool. Moreover, the questionnaire has been translated into seven other languages in an attempt to increase respondent rates and improve equity for people speaking and reading other languages than Swedish [31].

Based on relevant literature about PCC linked to the GPCC approach [9, 12, 13], we established four criteria for a questionnaire measuring patients’ perceptions of PCC: 1) the questionnaire should be operationalised to measure patients’ perceptions of PCC based on GPCCs three core routines and their ethical foundation; 2) the questionnaire should be generic, i.e. not tied to the setting, patient disease group or health care profession; 3) the questionnaire should be relatively short, with approximately 15 questions to minimise respondent burden; and 4) the questionnaire should have the potential to be used as an evaluation instrument (e.g., before and after measurement). In addition, we decided to use existing items in The National Patient Survey as a starting point in the development process.

**Fig. 1:**
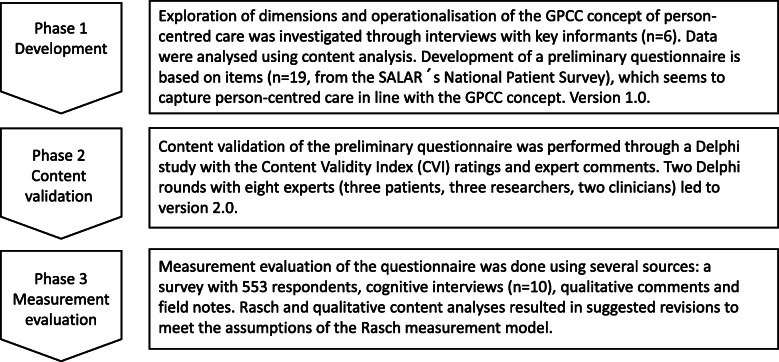
The three phases of questionnaire development: 1) Development, 2) Content validation, 3) Measurement evaluation

We conducted the study in agreement with recommendations for questionnaire development [32–34] using mixed methods in which the strengths of both qualitative and quantitative data analysis contributed to the interpretation of the results [35]. The process involved three phases: development of a questionnaire [32], content validation [36, 37] and evaluation of its measurement properties [38] (Fig. 1).

### Phase 1: development of questionnaire version 1.0

#### Key informant interviews

One of the research group members (CW) was well acquainted with PCC according to the GPCC conceptualisation. Interviews with key informants with knowledge of PCC in accordance with the GPCC approach were performed to complement this expertise and gain a broader view of the concept and its theoretical underpinnings. Six key informants, three researchers at the GPCC and three clinical experts from somatic and psychiatric care known for their experience of working in projects involving interventions based on the GPCC approach, were recommended by CW and purposively approached and recruited by email.

The interviews were conducted at different locations and times based on the informants’ preferences. One interview was conducted as a small focus group with three informants, one as a dyadic interview and one as an individual interview [39–41]. The first author, who led the interviews, had previous experience of interviewing and she had undergone interview training in a doctoral course. The last author had extensive knowledge of interviewing and acted as a note taker during the interviews. Interviews were semi-structured and covered the informants’ knowledge and thoughts on three overarching topics: PCC as a uni- or multidimensional construct, what items and activities to include to capture the full dimension(s) of PCC, and whether patients’ perceptions of PCC can be regarded as a product of the combined interaction of all health care professionals at a care unit or whether PCC needs to be operationalised for each health professional individually (see interview guide for key informants in Additional file 1).

Interviews ranged from 42 to 136 min (mean 81 min) in duration. Each interview was audio-recorded and transcribed. Qualitative content analysis with a deductive approach was used to systematically organise manifest data for making valid inferences used in the development phase of the questionnaire [42]. An unconstrained categorisation matrix was created using three main categories based on the three topics in the interview: Dimensionality (uni- vs. a multidimensional concept), what words and items may be used to operationalise PCC and patients’ perceptions of PCC according to the combined interaction of all HCPs vs. HCPs as individuals. The first and last author conducted the qualitative content analysis in Word. Initially, the first author identified all meaning units belonging to the predefined categories. Thereafter, in a constant dialog, the first and last author coded the identified meaning units based on their content and grouped the codes into subcategories. The result was discussed with all members of the research group.

#### Item selection from the National Patient survey

Based on the relevant literature and results from the qualitative content analysis the research group identified items in the National patient survey that captured the three core routines and an overarching ethical theme that spanned across all three actions. These items formed version 1.0 of the questionnaire.

### Phase 2: content validation of items and revision of the questionnaire (version 2.0)

Sixteen experts were purposively approached and asked to participate based on their knowledge of PCC from a GPCC conception. They were approached by email and informed about the purpose of the study, the timeline for participation, study procedures and a short introduction to Delphi and Content Validity Index (CVI) methodology [36, 43].

Eight experts consented to participate [44]: three patients from GPCC’s Person Council for patients [45], two health care practitioners and three researchers working in collaboration with the GPCC. Their experience of PCC varied from 1.5–16 years (median 5 years). The eight experts who declined participation cited uncertainty about having enough knowledge to contribute to the study (*n* = 1), or not being able to meet the timeline for participation (*n* = 7), (the Delphi study was carried out in June (2017) which is the end of the academic year in Sweden) as the primary reasons for their non-participation. The number of experts recruited were in line with recommendations on calculating CVI [44].

The Delphi study was conducted as a web survey with two rounds using a mixed-methods design with a) ratings based on the relevance of each item in the version 1.0 questionnaire on a scale from 1 to 4 and b) comments from the participants on each item for relevance, readability, comprehensiveness and suggestions of revisions, new items and dimensions in the questionnaire to make it complete. The response options were 1 = highly relevant, 2 = quite relevant (item needs some revision to be highly relevant), 3 = somewhat relevant (item needs major revision to be highly relevant), 4 = not relevant. The ratings were dichotomized into two groups: relevant (responses 1 and 2) and non-relevant (responses 3 and 4).

The item CVI (I-CVI) [37] was calculated for each item as the proportion of experts rating the item as relevant divided by the total number of experts. A minimum consensus level of 0.88 and positive comments consistent with the GPCC concept of PCC were set as a prerequisite for the retention of an item [44]. Comments on individual items and the questionnaire were analysed and discussed by the research group. Items were then added or rephrased based on the results from the I-CVI values and the participants’ comments [35] and returned to the participants for a new Delphi round. Experts obtained I-CVI summaries along with their ratings and anonymised comments from all experts and were asked to carry out a new validation of the revised and added items.

The research group conducted data analyses with I-CVI ratings and a Scale CVI calculated as an average (Ave) of the I-CVIs across the items (S-CVI/Ave) [43]. CVI analyses and qualitative comments informed a revised set of items and version of the questionnaire (i.e. questionnaire version 2.0).

### Phase 3: evaluation of measurement properties (version 2.0)

We used a mixed-methods design to evaluate the measurement properties of the questionnaire using the Rasch measurement model (RMM) and qualitative content analysis. Quantitative and qualitative data were first analysed and summarised separately.

The quantitative data were then merged with the qualitative data to elucidate the results further [35].

The RMM can be regarded as a blueprint for the basic criteria of fundamental measurement, providing necessary and sufficient means to transform ordinal counts of a latent trait into linear measures, given that the data fit the RMM [29, 46]. A key feature of the RMM is its property of invariance, i.e. items and persons responding to the items are estimated independently, thereby making it possible to compare the results from various contexts and populations.

#### Patient survey

Patients at six in- and outpatient care units in a region in Sweden were consecutively invited to respond to the questionnaire. The care units represented primary care, including rehabilitation and midwifery, psychiatric inpatient care for people with primarily depression and psychosis, geriatric in- and outpatient care at two hospitals and renal outpatient care. The recruiting process started in September 2017 and was carried out at two time points 1 year apart. Each recruitment phase was limited to 2 months for inpatients and outpatients, the exception being patients in primary care who were recruited by two people from the research group over 10 full days. Dates chosen for recruiting patients from primary care were based on information from the manager at the unit who confirmed these as being normal business days. Inclusion criteria were adults > 18 years and understanding the Swedish language or with access to a translator. Exclusion criteria were a diagnosis of dementia or unable to answer the questionnaire because of a severe psychiatric conditions or cognitive dysfunction. Patients were recruited and informed about the study either by a member from the research group or health care staff trained in the recruiting process. All participants were given verbal and written information about the study and completed version 2.0 of the questionnaire in paper format. At the end of each questionnaire, patients were encouraged to add comments in free text format about their perceptions of the health care at the unit where they were recruited. In addition, data on age, gender, education and occupation were collected. Patients who were unable to complete the questionnaire independently (e.g., because of reading difficulties or being unable to use their hands) were assisted in filling out the questionnaire by a member of the research group. Field notes were used for comments from those patients who required assistance to complete the questionnaire. Patients in outpatient care who were able to complete the questionnaire independently received the questionnaire and a pre-stamped envelope. One reminder was sent after 2 weeks to participants who had not yet responded.

Totally, 625 patients were asked to participate in the study. Of those 625 patients, 553 (88%) completed the questionnaire (participant characteristics are listed in Table 1), resulting in a sample size in line with recommendations for data analyses with the RMM [47]. Free-text comments were generated by 215 (39%) patients. Assistance to complete the questionnaire was given to 113 (20%) patients. Responses were treated as missing data and excluded from analyses if the “not applicable” response option was indicated, if two response options had been indicated for one item, or if a response was missing altogether. The response options for item 18 were “yes/no/not applicable” and respondents were asked to respond to items 19 and 20 only if they answered in the affirmative to item 18. Responses to items 19 and 20 were therefore treated as missing if patients had responded “no” to item 18, namely “Have you and your caregiver worked together to create a written plan for your future care and treatment?”.

**Table 1:** sociodemographic and clinical characteristics of the respondents (*n* = 553)

		*n*	%
Age			
Years, mean (SD)	66.7 (17.1)		
range, min/max	18–98		
Missing		11	2.0
Gender			
Female		261	47.2
Missing		9	1.6
Care			
Outpatient		387	70
Inpatient		166	30
Marital status			
Married/Cohabiting		305	55.2
Living apart		24	4.3
Living alone		208	37.6
Other		7	1.3
Missing		9	1.6
Educational level			
Comprehensive school		208	37.6
Upper secondary school		195	35.3
University		134	24.2
No former school		3	0.5
Missing		13	2.3
Occupation			
Working		95	17.2
Unemployed		18	3.3
Student		9	1.6
Pensioner		387	70
Other		24	4.3
Missing		20	3.6

The data were analysed with a focus on the following aspects: overall fit to the model, reliability, threshold functioning, individual item and person fit, targeting, differential item functioning, local dependency and dimensionality [38]. A summary of the statistical analyses and recommended set fit criteria for each analysis is presented in Table 2. RUMM2030 [55] was used for Rasch analysis using the partial credit model, which is suitable for polytomous response options [38, 56]. The Statistical Package for the Social Sciences (IBM SPSS version 26.0) was used to generate descriptive statistics. For in-depth information concerning Rasch measurement theory and data analyses, we refer to the methodological literature on these topics [38, 50, 51, 57, 58].

**Table 2:** Summary of the statistical analyses and set fit criteria applied for each analysis

Rasch statistic	Fit criteria
**Overall fit of items, mean and SD:** To what degree do observed item responses correlate with expected responses from the Rasch model?	Perfect fit = mean of 0 and SD of 1 Acceptable fit = SD < 1.5
**Overall fit of persons, mean and SD:** To what degree do observed person responses correlate with expected responses from the Rasch model?	Perfect fit = mean of 0 and SD of 1 Acceptable fit = SD < 1.5
**Item-trait interaction, chi-square probability value:** What is the probability that the overall responses fit the model?	Nonsignificant Bonferroni-adjusted probability value [38, 48] *
**Person separation index:** To what degree are item responses consistent across respondents?	Values ≥0.8 [49]
**Thresholds:** Do the response categories and thresholds work as intended, i.e. advancing monotonically, or are there any disordered thresholds?	Ordered thresholds [38]
**Individual item fit:** To what degree do the observed individual item responses correlate with expected responses from the Rasch model?	Fit residual = +/− 2.5 [50] Chi-square probability values nonsignificant with Bonferroni-adjusted probability value [38, 48] * Visual check of item characteristic curves in which the observed values should fit as closely as possible to the theoretical curve [38]
**Individual person fit:** To what degree do the observed individual responses correlate with expected responses from the Rasch model?	Fit residual = +/− 2.5 [50]
**Targeting:** To what degree are items targeted to the persons in the sample?	Mean location score for the persons should be close to the mean value of zero set for the items [51]
**Differential item functioning:** Does any item deviate from the a-priori requirement of invariance across groups for gender, age and care?	Nonsignificant Bonferroni-adjusted probability value [38, 48] *
**Local dependency:** Does any item show dependency on a response to another item?	No positive residual correlations > 0.2 above the average residual correlations across all items [52]
**Dimensionality:** To what degree does the questionnaire measure one single dimension?	The proportion of t tests reaching significance should not exceed 5% in the independent t test protocol [53, 54]

^*^ Bonferroni adjustments for multiple null hypothesis testing were applied with the alpha level of significance set at 0.05 [48]

### Cognitive interviews

Cognitive interviews with patients were undertaken to explore a) response options and anchors in the questionnaire, b) overall content, c) responses related to the Rasch analysis and d) comprehension and interpretation of the items. Inclusion and exclusion criteria were the same as for the patient survey. We recruited a purposive sample based on subject variation across age, gender and type of unit [59]. Ten patients (six females) aged from 54 to 85 years (mean 71.5 years) were recruited from four in- and outpatient care units. A pragmatic approach was used regarding the number of patients recruited for cognitive interviews. As a large number of patients (*n* = 113) had contributed to the response process when they were assisted to complete the questionnaire by a member of the research team, ten patients were judged as a reasonable number to support data based on the field notes and free-text comments. Interviews lasted from 16 to 55 min (mean 38 min) and took place in a location at the health care unit chosen by the patients. Patients were encouraged to think aloud as they completed the questionnaire. Retrospective verbal probes were used at the end of each cognitive interview to clarify the patients’ comprehension of the questionnaire [60]. The first author conducted the interviews and used a protocol based on the questionnaire (see Additional file 2) to note comments from each patient while he or she responded to the questionnaire. All interviews were audio-recorded and transcribed.

### Qualitative data analysis

Transcribed data from cognitive interviews, together with field notes and free text comments from the survey were analysed in Word using deductive content analysis, to systematically organise manifest data for making valid inferences regarding participants responses to the questionnaire [42]. The analysis was performed by the first author in close collaboration with the last author. An unconstrained matrix was created with the main categories similar to the four topics for the cognitive interviews. Meaning units in the text identified as belonging to one of the main categories were included in the analysis and coded based on their content. To describe each of the main categories’ codes were grouped into subcategories. The results were discussed with all members of the research group.

In a mixed-methods approach, findings from content and Rasch analyses were converged to elucidate and explain findings and to inform revisions of the questionnaire.

## Results

### Phase 1: development of a questionnaire version 1.0

Results from the qualitative content analysis, based on key informant interviews, are displayed in Additional file 3 and presented below according to the three pre-defined main categories: a) Dimensionality (uni- vs. multidimensional concept), b) Operationalisation and c) Patients’ perceptions of PCC based on the combined interactions with all HCPs vs. HCPs as individuals.

The informants stressed the underlying ethical approach and that PCC as such could be regarded as a *unidimensional concept.* The three independent but interlinked core routines meet the challenge of securing a philosophical and ethical approach that is concrete, so it is understandable and can be acted upon within a health care context. This was related by one informant in the focus group as: *It’s not just about these three actions, not that they are three separate things, but more like we discussed in the beginning that it is the same dimension.*

Moreover, informants described how the *operationalisation* of PCC needed to be based on patients’ perceptions of HCPs treating them as an integral part in the care process. Such an approach implies that care should be co-created between HCPs and patients with a focus on the patient’s unique knowledge and resources. Items can be based on the three core routines but should reflect the underlying ethical approach (e.g., partnership based on mutual respect, trust and reliance). The informant from the individual interview described and gave an example of how sharing of information between a health care professional and a patient could be operationalised based on PCC. *Information is really important, but it can easily become a one-way communication, we have informed the patient, but it is important that there is two-way communication. When I give information to the patient it is important that the patient gets the question “what do you think?” and “is it ok?”. If we do it this way, then the patient can feel that they were questioned and can be a part of making decisions.*

PCC was discussed within a health care context as something that should permeate the whole culture of the workplace, including patients’ perceptions of PCC based on *the combined interactions with all health care professionals*. Informants described that all personnel should be part of a change towards more PCC to ensure continuity and equity for all patients. This perspective promotes operationalising PCC from a group perspective in which staff members work together with patients to foster a PCC environment perceived by patients as respectful, equitable and inclusive. Informants also saw PCC from an individual perspective in which each HCP needs to find, through experience and self-reflection, his or her way to understand the ethical underpinnings of PCC. One informant in the focus group underscored this individualistic perspective by stating: *It goes without saying that some people just have it and we can’t just say that everybody just has it.*

Based on the relevant literature and findings from the qualitative content analysis we decided to operationalise PCC as a) a broad overarching unidimensional concept focusing on the co-creation of care in the meeting between HCPs and patients; b) a questionnaire with items targeting GPCC’s three core routines, as well as items thought to capture the ethical approach in a broader sense; and c) a questionnaire capturing patients’ perceived level of PCC based on either their interactions with one HCP (i.e. in an outpatient context where patients have one HCP involved in their care) or based on their combined interactions with all HCPs at a specific unit.

We identified items in SALAR’s existing item pool of 32 items that corresponded to the findings in the qualitative content analysis (for details see Additional files 3 and 4). The selected items (*n* = 19) in the preliminary questionnaire were considered to capture central aspects of PCC in accordance with the GPCC approach. The response scale, ranging from 1 to 5 with anchors representing “no, not at all” to “yes, completely” and the possibility to respond “not applicable” to each question, was retained from the National Patient Survey.

### Phase 2: content validation of items and revision of questionnaire to version 2.0

After the first Delphi round, 17 of 19 items achieved I-CVI ratings of 0.88 or 1.0. Comments from experts on specific items were primarily about changing the wording. The words *together with, encouraged, invited, in collaboration with* were proposed as opposed to the word *get,* which was described as reducing patients to passive reciprocates of care. Examples from the reviewing process are shown in Additional file 5. Suggestions to add items were largely based on capturing the partnership between HCPs and patients, acknowledging patients’ resources and confirming the patient as an active partner in their health care process. Three items were described as problematic because they are double-barrelled, making the items difficult to interpret. *Compassionate and sympathetic, respect and dignity, compassion and care* were phrases giving rise to new suggestions such as *respect and compassion*. A decision was reached to limit each item to one term to decrease multiple interpretations.

After the second Delphi round, 24 of the 25 items obtained CVI ratings of 0.88 or 1.0 (Additional file 4). S-CVI/Ave reached .95, well above a recommended consensus level of 0.90 [37, 43]. Although CVI analyses showed excellent consensus levels between experts, the participants still suggested revisions to some of the items as well as preferences for some items over others. I-CVI ratings and qualitative comments were reviewed and prompted a revised set of items (*n* = 20), which led to the second version of the questionnaire (version 2.0).

### Phase 3: evaluation of measurement properties (version 2.0)

#### Responses across respondents

Of 553 respondents, 87 had extreme scores, i.e. they responded consistently throughout the questionnaire to the highest (*n* = 86) or lowest (*n* = 1) response alternatives for all items. Respondents with extreme scores were excluded from estimation of item statistics by default in RUMM, as they cannot contribute any additional information about how items are situated as to “difficulty” estimates on the common logit score. Thus, 466 respondents were included in the estimation of item statistics. All items had missing responses, with most belonging to the response option “not applicable”. The number of missing responses for each item ranged from 9 to 346. Items 19 and 20 had the largest number of missing responses with 345 and 346, respectively. The missing responses for these two items were partly due to responses being treated as missing if the respondent replied *no* to item 18 but then went on and replied to items 19 and 20. Two other items (13 and 18) also had a substantial number of missing responses (item 13, *n* = 157 and item 18, *n* = 153).

The qualitative analysis of the response options and anchors provided some explanations for the number of missing responses. For item 18 (“Have you and your caregiver worked together to create a written plan for your future care and treatment?”), respondents were asked why they responded to items 19 and 20, even though they had responded *no* to item 18. Some respondents said that they did not have a written health care plan but had agreed verbally on a plan for the future and thereby chose to respond to items 19 and 20. Data from field notes revealed another cause for concern. Respondents who received assistance to complete the questionnaire were sometimes known by the members of the research group to have a written health care plan. However, some patients were still hesitant to endorse item 18, reasoning that they were unsure about what a written plan was, whether they had ever been involved in developing such a plan, or even if such a plan existed.

When responding to item 13 (“Were your relatives given the opportunity to participate in your care and treatment to the extent you wished?”), many respondents chose the response option *not applicable* because they did not have any relatives or did not want relatives to participate in their care.

#### The overall fit of items and respondents

A summary of the fit statistics, indicating to what degree the questionnaire as a whole fits the assumptions of the RMM, is shown in Table 3 in three versions. The first version showed some misfit to the expectations of the RMM. This misfit was indicated as a small and significant summary chi-square value < 0.001, and a summary item residual standard deviation of 2.413. The observed summary person residual standard deviation was high, with a value of 1.464 but still within what was set as an a-priori acceptable fit value. Further tests to evaluate a unidimensional measure by identifying negatively and positively loading items in a principal component analysis were separately performed to yield estimations of a person’s location. These two estimations were then compared for each person by conducting a series of independent t tests, which showed significantly different person estimates in 15 patients (3.35% of the cases) after omitting extreme cases. The independent t tests were within the set fit criteria of < 5%, indicating that there was no evidence of multidimensionality among the items.

**Table 3:** Summary of Rasch analyses assessing the overall model fit performed in three stages along with the iterative revisions

Analysis	Item residual		Person residual		Chi-square interaction			Psi	T test *n* (%, CI)
	Mean	SD	Mean	SD	Value	DF	*P*		
Original version	0.355	2.413	−0.296	1.464	371.1	160	**< 0.001**	0.84	15 (3.35%, 1.8–4.8%)
Thresholds ordered	−0.052	1.880	−0.347	1.416	330.5	160	**< 0.001**	0.85	16 (3.57%, 2–5.1%)
Items 13, 18 deleted	0.322	1.561	−0.383	1.521	180.3	144	0.022	0.85	16 (3.80%, 2.2–5.4%)

Values highlighted in bold in the probability column indicate statistical significance at the 0.05 level after Bonferroni adjustment for the original version, with thresholds ordered set at 0.0025 for 20 items and with two items deleted set at 0.0027 for 18 items

Independent t tests were performed with eight items divided into two subsets representing most diversity along a continuum. Each subset included > 15 thresholds for the original version and > 12 thresholds for the version with thresholds ordered and with two items deleted

*DF* degrees of freedom

*P* probability value

*Psi* Person separation index reported with extrapolated person values included

*CI* confidence interval reported as lowest to highest

In general, the qualitative analyses (Additional file 6) indicated that the patients perceived the overall content of the questionnaire as valuable and suitable in a health care context. The questionnaire was perceived to capture and depict a part of health care that seemed pertinent to patients’ recognition of high-quality health care, namely how they were approached and treated by HCPs explained by one respondent saying: *The questions are quite good, they capture what it’s about, you get a picture of how things are in health care.*

#### Reliability

Evaluation of the internal consistency of the questionnaire with 20 items and five response categories for 19 items and two response categories for one item showed a Person separation index (Psi) value of 0.84 (including extrapolated person values).

#### Response category functioning

**Fig. 2:**
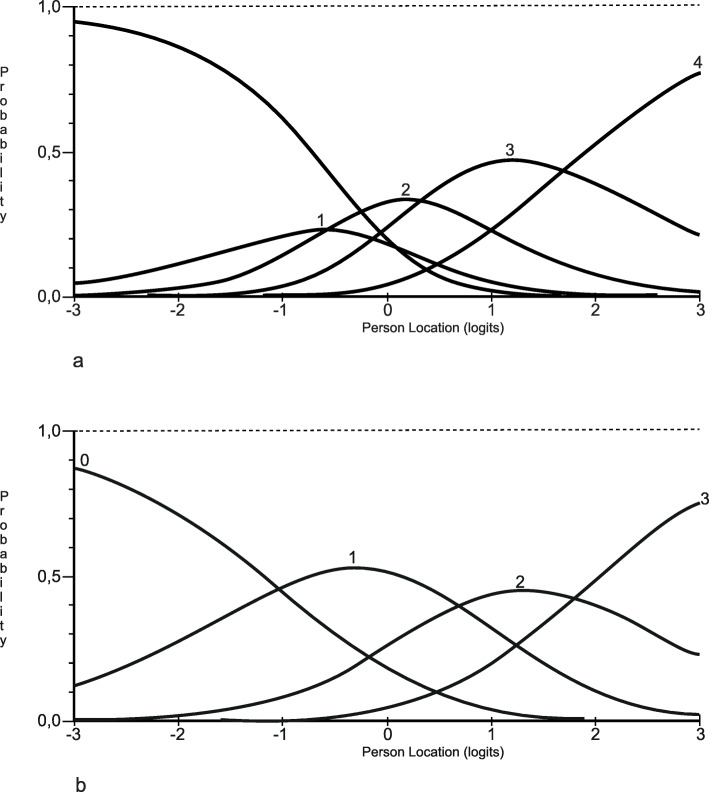
Category probability curves for item 2, before and after thresholds had been ordered. Panel a shows disordered thresholds and panel b ordered thresholds after categories 0 and 1 had been collapsed into one category

The Rasch analysis indicated that 14 of 20 items had disordered thresholds. Before further analyses were conducted, we rescored items with disordered thresholds. The disordered thresholds were mainly found in the lower end of the response categories (1 and 2), which also coincided with low response frequencies. The rescoring option lending the best fit to the model, i.e. evaluation of ordered thresholds, fit residuals and item chi-square probability values was chosen for each item. Fourteen items with disordered thresholds were rescored into four categories and one item (item 13) was rescored into three categories. An example of the category probability curve for item 2 (“Did you and the staff discuss how your state of health/your illness can affect your everyday life?”) before and after thresholds have been ordered is shown in Fig. 2. After the disordered thresholds had been resolved, the summary fit statistics still showed misfit according to the expectations of the RMM. This misfit was indicated as a low and significant summary Chi-Square value (< 0.001) and a summary item residual standard deviation of 1.88, indicating an improved fit compared with the original version but still higher than what had been set as the a-priori fit (Table 3).

The qualitative analysis corroborated and explained some of the results from the Rasch analysis for the response category functioning. First, there were some conflicting views on the response options for the items. Some respondents perceived difficulty in choosing between the response options: as one respondent said, “*Many questions are difficult to respond to. This 1, 2, 3, 4, 5 … it’s like … you could write anything.”* Other respondents wondered what each number represented and preferred labels for each response option, whereas others had no problems, stating that they were used to these kinds of response options.

Second, some respondents who were part of care that involved several health care professionals identified difficulties in using the single response scale to rate their interactions with all health care professionals, especially for those who had mixed care experiences. Some respondents described co-creation of care and the meeting between health care professionals and patients as relying on individual interactions in which the HCPs were perceived to interact differently with the respondents. One respondent described this difficulty saying, “*It’s difficult to answer the questions and think about all the staff. There’s a big difference between how different staff members treat you.”.* Of note, the discrepancies between HCPs as perceived by the patients, were not tied to the staff members vocational role. Thus, for example, making an overall evaluation of interactions within the group of working nurses was just as problematic as making comparisons between nurses and medical doctors. The cognitive interviews indicated that respondents solved this issue by making an overall evaluation across all health care staff when they selected a response option. One staff member alone could thereby act to increase or decrease a patient’s evaluation of perceived PCC.

#### Individual item fit (after items had been rescored to obtain ordered thresholds)

As shown in Tables 4, 16 of 20 items exhibited fit residuals within the recommended range (+ − 2.5); of those four items with fit residuals outside the recommended range, two had significant chi-square values (items 16 and 18). Graphical inspection of item characteristic curves (ICCs) for these items showed deviations from the model in accordance with fit residuals.

**Table 4:** Summary of the item and fit statistics for items in the initial and revised versions

Items	Initial version with 20 items					Missing	Revised version with 18 items				
No. Item	Location	SE	Fitresid	Chi sq.	Prob		Location	SE	Fitresid	Chi sq	Prob
1. Listen to your experiences of health and illness	− 0.550	0.076	− 0.577	8.212	0.413	17 (3)	−0.465	0.077	0.162	5.851	0.664
2. Discuss how health and illness affect ADL	0.489	0.068	0.539	4.349	0.824	39 (7)	0.612	0.070	1.535	1.759	0.988
3. Encouraged to ask questions	0.556	0.066	1.765	15.234	0.055	36 (7)	0.678	0.067	**2.985**	21.446	0.006
4. Get responses that you understand	−0.610	0.070	−1.167	6.191	0.626	18 (3)	−0.539	0.071	−0.574	6.104	0.636
5. Enough information about care and treatment	−0.336	0.072	−0.998	13.295	0.102	12 (2)	−0.242	0.074	−0.346	11.392	0.180
6. Come to an agreement on the next step in care	−0.083	0.072	0.273	4.763	0.783	57 (10)	0.023	0.073	1.495	5.525	0.700
7. Participate in care-related decisions	0.261	0.070	−0.830	8.650	0.373	49 (9)	0.373	0.071	−0.036	3.145	0.925
8. Important ADLs were considered in planning	0.179	0.061	−1.330	16.214	0.039	56 (10)	0.285	0.062	−0.370	14.087	0.080
9. Important goals set for the planning of care	0.564	0.071	−1.705	9.494	0.302	79 (14)	0.691	0.073	−0.677	9.417	0.308
10. Coordination of contacts within care	0.026	0.063	1.413	12.524	0.129	97 (18)	0.121	0.065	**2.546**	9.851	0.276
11. Discuss what you can do for yourself	0.174	0.070	−0.105	12.336	0.137	46 (8)	0.284	0.071	1.078	10.606	0.225
12. Resources acknowledged and utilised	0.121	0.062	−1.190	11.295	0.186	50 (9)	0.227	0.063	−0.401	17.046	0.030
13. Opportunity for relatives to participate	−0.302	0.098	**3.941**	26.325	**< 0.001**	157 (28)	deleted				
14. Opportunity to express when concerned and anxious	−0.140	0.073	−0.143	8.833	0.357	67 (12)	−0.048	0.075	0.764	10.537	0.229
15. Feel as an equal person	−0.214	0.071	1.117	8.407	0.395	13 (2)	−0.117	0.072	1.991	6.329	0.610
16. Trust in the staff/caregiver	−0.968	0.082	**−3.093**	28.725	**< 0.001**	10 (2)	−0.911	0.083	**−2.742**	22.491	0.004
17. Treated with respect	−1.047	0.083	**−3.080**	21.371	0.006	9 (2)	−0.986	0.085	**−2.726**	15.126	0.057
18. A plan written together with the staff/caregiver	2.030	0.128	**3.934**	100.433	**< 0.001**	153 (28)	deleted				
19. Participate in the development of the plan	0.153	0.102	−0.583	9.434	0.307	346 (63)	0.230	0.104	0.096	6.235	0.621
20. Understand the written plan	−0.303	0.122	0.773	4.434	0.816	345 (62)	−0.214	0.124	1.206	3.353	0.910

Analyses have been performed with patients divided into nine class intervals with about 50 persons in each interval for all items, except four items 13, 18, 19 and 20

Items 13 and 18 have approximately 40 persons in each class interval and items 19 and 20 have about 19 persons (class intervals are based on groups within the sample with similar perceived levels of PCC)

Values highlighted in bold in the fit residual column show items with fit residuals outside the recommended range of + − 2.5

Values highlighted in bold in the probability column indicate statistical significance at the 0.05 level following Bonferroni adjustment for the initial version set at 0.002 for 20 items and 0.003 for the revised version with 18 items

Missing responses represented as *n* (%)

A closer examination of fit residuals and ICCs showed that items 16 and 17 had large negative fit residuals, suggesting a possible local response dependency. In contrast, items 19 and 20 had large positive fit residuals, suggesting that these items may be measuring something different than the underlying common trait. Local dependency was examined by checking for positive residual correlations > 0.2 above the average residual correlations across all items. The residual correlations showed a correlation of 0.579 between items 16 and 17 and 0.405 between items 19 and 20. Local dependency was examined further by creating two subtests of these item pairs. Subtests were carried out separately starting with items 16 and 17. The results indicated a negligible change in reliability estimates, which dropped from 0.85 to 0.84 for the first subtest and remained the same (0.85) for the second subtest (including extrapolated values).

The qualitative analysis of comprehension and interpretation of the items indicated that the respondents generally seemed to understand the items as intended. However, the wording in some of the items, e.g. item 5 (“Did you receive *enough* information about your care and treatment?”) and item 7 (“Did you participate, *to the extent you wished*, in decision making about your care and treatment?”) led to different comments from the respondents, such as, “*how would I know what is enough* and *what can I get?”* Furthermore, concerning item 12 (“Were your *resources* like your will, drive, knowledge and physical capacity, utilised concerning your care and treatment?”), respondents noted how they had difficulties interpreting the word *resources* concerning their situation.

Moreover, items targeting increased participation and making shared decisions were often viewed from two opposing perspectives which is exemplified in item 7 (“Did you participate to the extent you wished in decision making about your care and treatment?). Some respondents reasoned that the item was strange in the sense that it is the HCPs job (or responsibility) and not theirs to make decisions about health care. In contrast, others felt this question was highly relevant, as illustrated by one respondent’s comment: “*There are a lot of things in health care that the staff members take for granted. I want to be a part of making decisions for myself. After all, it’s about me and my body”.*

#### Individual person fit

Some respondents (*n* = 43) were identified as not meeting the expected response profile of the RMM. A closer analysis of these respondents, when compared with the rest of the sample, revealed no significant (*p* > 0.05) differences in age, gender, and care that could explain the misfit.

#### Targeting

**Fig. 3:**
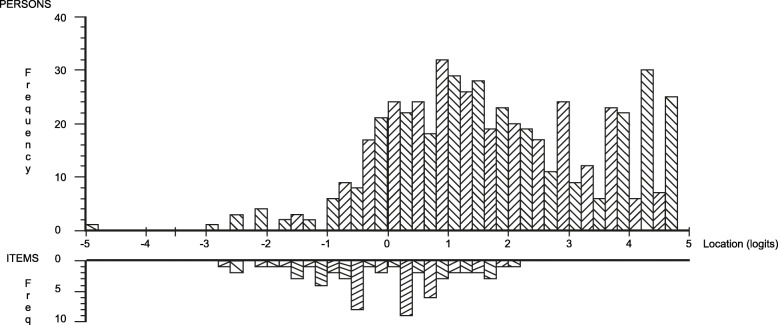
Targeting depicted in the Person-item Distribution Map. Patient locations are displayed at the top half of the graph and item thresholds at the bottom half of the graph

The distribution of item thresholds displayed an even distribution spanning across five logits (− 3 to 2), representing increasing levels of patients’ perceived PCC. The distribution of persons, in turn, were spread across eight logits (− 3 to 5) with a mean of 1.7 (SD 1.59), indicating that items failed to capture all the respondents’ perceived levels of PCC. The mistargeting was particularly evident in the higher end of the scale, where items were unable to capture higher levels of perceived PCC. Thus, on average, patients reported perceived PCC levels of 1.7 logits above that represented by the items in the questionnaire, which is always set at 0 as a function of the model (Fig. 3).

The skewed targeting can be partly explained by the results from the content analysis. First, many respondents reported that care at the units was excellent and superior to what they usually experienced. Respondents described how their responses had to be seen in the light of that specific unit where they were treated and that responses would have been different if they had based their care experiences at other units. One respondent phrased this attitude as “*I want the staff to understand that they are an important support for me. They are positive; they explain; they don’t just do things over my head because at other places they don’t listen to me at all”.* Second, a contrasting finding from the field note data indicated that some patients seemed unwilling to endorse low response options even when these were consistent with their care perceptions. This contrasting viewpoint is exemplified by one respondent who needed assistance with completing the questionnaire and said that he had not discussed his care in terms of those found in item 11 (“Did you and the staff discuss what you can do yourself to improve your state of health/your illness?”). However, the respondent were steadfast in his belief that the highest response option should be given, explaining, “*I don’t want you to indicate a low response alternative; indicate the highest [response] because I am delighted with my care and I don’t want to give any low responses*.” Third, patients sometimes chose a non-applicable response option to bypass a low response option. This strategy was applied by patients who sometimes chose the *non-applicable* response option when confronted with an item that captured care they found hard to confirm with a high response option.

#### Differential item functioning (DIF)

Patients did not differ in their responses across the items for gender, age (divided into two groups by the median of 71 years) or care setting (inpatient *n* = 166, outpatient *n* = 387), i.e. there was no uniform or non-uniform DIF across items for gender, age or care after a Bonferroni adjustment of 0.00083 was applied.

#### Revisions of the questionnaire

**Fig. 4:**
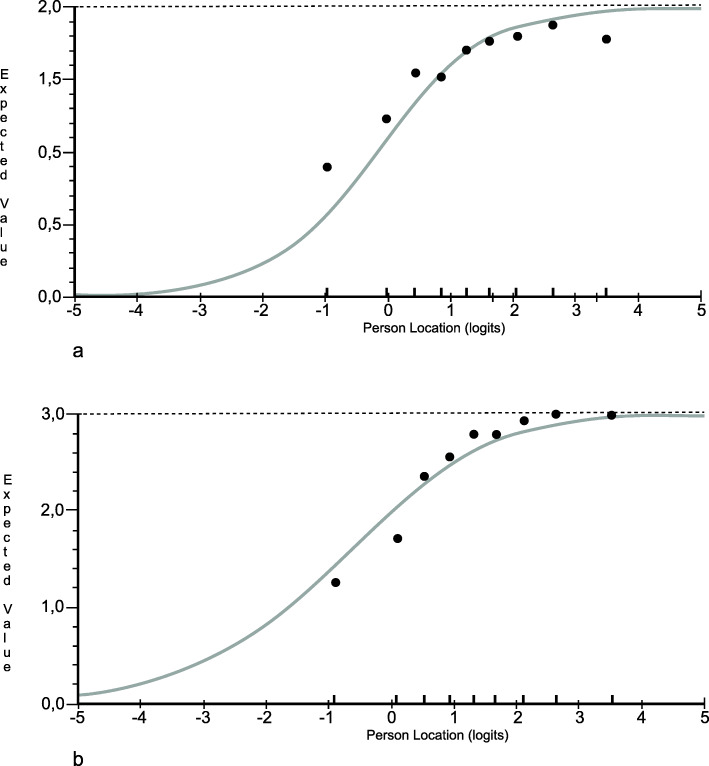
Item characteristic curves of items 13 and 16 (when item 18 has been deleted) The grey curves represent the expected item responses and the black dots the observed item responses with patients grouped into nine class intervals based on similar person locations. Panel a shows item 13 with a large positive residual (4.753) and dots that deviate from the expected curve creating a flatter pattern. Panel b shows item 16 with an opposite pattern with a large negative residual (−2.832) and a pattern that is steeper than the expected curve

Findings from the Rasch and qualitative analyses guided further revisions of the questionnaire. Items 18 and 13 were considered the most troublesome. Both items had large positive fit residuals (suggesting multidimensionality) and missing responses (suggesting low relevance). Moreover, these two items can perhaps be viewed to conceptually represent something that is contingent on routines in a workplace and external to the patients (i.e. documentation of plans and relatives being invited to be part of patients care). Items 18 and 13 may therefore not work as intended with the remaining items in the questionnaire. These items were subsequently deleted sequentially, starting with item 18, which was identified as most misfitting. After item 18 was deleted, all items showed acceptable fit to the model, except for items 13 and 16, where item 13 showed the worst fit with a large positive fit residual and significant probability value. The values were corroborated by ICCs showing graphical deviations from the expected pattern, suggesting that item 13 may measure something different from the rest of the items (Fig. 4).

When item 13 was deleted, four items still had fit residuals outside the a-priori fit criteria, but these deviations were not statistically significant (Table 4). The overall fit of the items and persons in the model was improved compared with the original dataset with a nonsignificant overall item-trait interaction chi-square statistic (Table 3). Even though items showed acceptable fit to the model, a slight deviation from the a-priori acceptable fit values was seen for both items and persons. Repeated independent t tests remained within the set fit criteria. The psi was slightly improved with a value of 0.85 compared with 0.84 (including extrapolated person values) [51]. In summary, the overall fit of the items improved after the changes were made to the original dataset (Table 4).

## Discussion

We have reported on the development of a generic questionnaire measuring patients’ perceptions of PCC. We also evaluated the questionnaire’s content validity and measurement properties. The study was carried out in three phases, each following on from the other in line with recommendations for questionnaire development [33, 34].

Results from phase one were essential to gain a thorough knowledge of PCC and increase content and construct validity. Interviews with key informants underscored the importance of viewing the GPCC conceptualisation of PCC as something more than just the three core routines advanced in the Centre’s position paper in 2011 [9]. Informants were uncompromising in their belief that PCC is an overarching ethical approach that defines how patients and HCPs relate to one another and as such is a unidimensional construct. The notion of placing emphasis and priority on the philosophical and ethical underpinnings of PCC when it is operationalised in everyday clinical practice is in line with results from an interview study based on clinician-researchers experiences from implementing PCC in various settings [61]. The clinician-researchers underscored that a change in HCPs underlying perception and recognition of each patient as a person, is just as important as the three core routines in GPCC’s ethical approach [61]. Similarly, McCormack, who has conducted extensive work on PCC [7], noted that a prerequisite for achieving PCC in clinical practice is to make sure that the philosophical underpinnings of personhood are understood and embedded in all PCC approaches [1]. Perhaps more importantly, patients seem to regard ethical aspects in their health care as essential for their care experience [62]. Patients in an interview study appeared to place more value on informal aspects of the partnership with HCPs , such as human connectedness, before formal aspects like goal setting and documentation [62].

In phase two consensus levels were high between experts in the CVI ratings first round and even higher by the second round. Overall, there was an association between the qualitative data from the key informants in phase 1 and the experts in phase 2. Triangulating results from different data sources strengthened the content and construct validity of the questionnaire. We argue that mixing CVI ratings with qualitative comments produced richer data, which led to a more in-depth characterisation of the latent construct that is operationalised and validated. The content of the items in the developed questionnaire share many similarities with the WHOs core principles of People-centred care [2] and the newly published European Standard for PCC [14]. These similarities strengthen the generalisability of the results in phase two.

In phase three problems from a fit statistic and qualitative perspective were identified in items 13 and 18. Item 13, concerning relatives’ opportunity to participate in patient care, seemed to represent something different from most of the other items in the questionnaire. Relatives represent a valuable source of support in PCC and can serve as a support or proxy for patients who are unable to speak for themselves [12]. However, the respondents who participated in this study were able to do so without help from their relatives. For this reason, this item was perhaps not relevant to these respondents. This misfit is supported by the results in two previous studies where items based on relatives’ involvement were subsequently removed to meet the assumptions of the RMM [20, 63]. PCC is still a developing concept and the aspect of relatives may likely be a dimension that needs to be measured on its own.

Item 18 also showed large significant fit residuals and was identified as problematic in the qualitative analysis. While some patients were unaware that they had a written plan, others described that this was not an important aspect of their care and treatment. This observation is in line with another qualitative study in which the HCPs viewed the plan as an essential part of PCC compared with the patients who acknowledged other aspects in health care as more meaningful [62]. Item 18 was also seen as misguiding in the sense that some patients were known to have a plan but were themselves unsure of its existence. This item should probably be revised or supplemented with an explanation or as a descriptive item identifying the number of respondents who are aware that they have a written plan.

After a revision of thresholds and deletion of items 13 and 18, the resulting fit of the final questionnaire is reasonable, albeit far from perfect. The immediate problem is the miss targeting between items and persons, i.e. creating a ceiling effect. The results from the qualitative analysis indicate that recruiting bias might be a potential cause for the targeting problem. The patients perceived that the health care units in the current study adhered to high levels of PCC compared with their experience of care at other settings. However, other studies have reported that targeting problems are common in surveys based on patients’ self-ratings of perceived care [20, 63]. To improve targeting in further versions of the questionnaire it would be desirable to include items representing higher levels of PCC, as well as to impose changes to the response anchors. Future studies should include testing the questionnaire in other settings assumed to have lower levels of PCC to validate whether such data have the potential to improve targeting and be used as an evaluation measure in longitudinal studies. Moreover, the content analysis conveyed that some respondents found it difficult to interpret the response scale when responding to the items with more than one HCP in mind. This difficulty warrants further research to clarify possible inconsistencies in response patterns based on cognitive load and interpretations of the response scale. We still prefer to use the questionnaire as a measure targeting patients’ perceptions of PCC as a product of the single interaction with one HCP or as the combined interaction with all HCPs in a health care setting. Health care in an inpatient setting is, by nature, based on staff working in shifts in a continuously changing workplace. Moreover, care is often based on interdisciplinary teamwork in which the patient and different care providers may take somewhat different roles and tasks in relation to the co-creation of care. This dualistic position is in accordance with the information advanced by the key informants in phase 1, where each individual and HCP need to cooperate to create a PCC environment. In addition, further evaluations of the questionnaires’ generic properties should be performed in other populations, including younger age groups, patients representing culturally diverse individuals and various disease groups. For now, the questionnaire can be used to set a minimum standard for benchmarking PCC and as a tool to evaluate patients’ perceptions of PCC alongside other patient-reported outcomes.

Throughout the process we strived to include patients, however the fact that no patients were recruited as key informants in phase one or engaged to choose items from the National Patient Survey, can be considered a weakness. We argue that the aim for phase one was to gain a deeper knowledge of the philosophical and theoretical underpinnings of GPCC as a concept, leading us to include only researchers and clinicians as key informants in that stage of the study. However, one needs to consider that GPCC is continuously working together with patients to develop the approach and understanding of PCC [45]. Furthermore, items in the National Patient Survey were developed in conjunction with patients to strengthen relevance, clarity, and readability [30].

This study used several methods to collect and analyse data. PCC is still a developing concept and mixed methods have been a core methodological focus in this study, which is also widely recommended in the literature on questionnaire development [32–34, 64]. Results from the Rasch analyses had not been possible to interpret in such detail without performing concurrent qualitative data analyses. The results will be highly useful to guide future revisions of the questionnaire and increase the understanding of PCC.

## Conclusion

We have reported the development of a proposed generic questionnaire measuring patients’ perceptions of PCC, the Generic Person-Centred Care Questionnaire (GPCCQ). We also evaluated the content validity and measurement properties of the questionnaire. The study applied several methods to collect and analyse qualitative and quantitative data to explain, extend and validate the questionnaire’s measurement properties. When disordered thresholds were resolved and two misfitting items deleted, data from the questionnaire were able to meet the requirements for measurement assumed by the RMM. Although the requirements were met, there is still a problem with targeting that needs to be addressed in future studies. However, for the time being, we consider the questionnaire merits as a measure of quality and benchmarking of PCC.

## Supplementary information

**Additional file 1.** Interview guide key informants. Interview guide for key informants in phase one.

**Additional file 2.** Interview protocol cognitive interviews. Interview guide for cognitive interviews with patients in phase three.

**Additional file 3.** Content analysis key informants. Subcategories and codes generated using an unconstrained matrix with three pre-defined main categories.

**Additional file 4.** Item conceptualisation and I-CVI 1st and 2nd round. Operationalisation of items intended to present PCC in line with GPCC’s concept and I-CVI calculations for first and second Delphi round.

**Additional file 5.** Examples of the Delphi process. Examples from the Delphi process, including I-CVI values and comments from experts and resulting revisions.

**Additional file 6.** Content analysis cognitive interviews, free-text comments and field notes. Subcategories and codes generated using an unconstrained matrix with four pre-defined main categories.

## Data Availability

The datasets used or analysed during this study are available from the corresponding author on reasonable request.

## Abbreviations

**WHO**: The World Health Organization
**PCC**: Person-centred care
**GPCC**: The Gothenburg University Centre for Person-centred Care
**SALAR**: The Swedish Association of Local Authorities and Regions
**RMM**: Rasch Measurement Model
**CVI**: Content Validity Index
