# Supplementary material for: Development and evaluation of the measurement properties of a generic questionnaire measuring patient perceptions of person-centred care
Source: BMC Health Serv Res. 2020 Oct 20;20:960. doi: 10.1186/s12913-020-05770-w (PMC7574493; doi:10.1186/s12913-020-05770-w)
Supplement: Supplementary file 1 — Additional file 1. Interview guide key informants. Interview guide for key informants in phase one. [file 12913_2020_5770_MOESM1_ESM.pdf]

Additional file 1. Interview guide key informants. Interview guide for key informants in phase one.

Interview guide for key informants

Presentation and background:
Introduction of myself and my supervisor. Previous work in clinical care, research and positions held at the university.
Project background: We aim to investigate the implementation process of person-centred care (PCC) at six different units in a region in Sweden. Several measures will be developed and used to capture person centred care as part of evaluating the implementation process. These measures will be based and analysed using the Rasch-model. An assumption when we use The Rasch-model is that all questions in a questionnaire must measure the same underlying construct or phenomena if we want to use the sum score. We have contacted you to ask for your assistance in helping us better understand GPCC's perspective on PCC in order to capture a fair and as complete picture of the concept as possible.

We need your help in defining PCC and creating clear criteria for what an instrument should contain in order to reflect and capture as correct a picture as possible of PCC.

Questions:

1. How is your view on the concept Person centred care from a dimensional perspective? Can it be regarded as **one single dimension or is it made up of multi-dimensions?** To what degree can the three concepts of PCC -the narrative, partnership, and documentation be assumed to belong to the **same dimension**? Can questions posed in the different parts be assumed to be measuring the same thing? Does the questionnaire need to be structured in one, two, or three different parts based on the narrative, partnership, and documentation?
2. **Which abilities/behaviours** form the core of PCC and need to be included in an instrument in order to capture as thorough and complete picture of the concept as possible?
3. The instruments are thought to include all the categories of healthcare workers thought to in some way be actively involved in patient care, e.g. assistant nurses, registered nurses, physiotherapists, occupational therapists, physicians, etc. Are there any differences that we must take into consideration regarding **how the different staff and vocational groups work with PCC**?

Probes: Do you have anything more to add? Can you give an example? Have I understood you correctly/do you mean that… [moderator summarises the content of the discussion]?
