# Supplementary material for: Development and evaluation of the measurement properties of a generic questionnaire measuring patient perceptions of person-centred care
Source: BMC Health Serv Res. 2020 Oct 20;20:960. doi: 10.1186/s12913-020-05770-w (PMC7574493; doi:10.1186/s12913-020-05770-w)
Supplement: Supplementary file 2 — Additional file 2. Interview protocol cognitive interviews. Interview guide for cognitive interviews with patients in phase three. [file 12913_2020_5770_MOESM2_ESM.pdf]

Additional file 2. Interview protocol cognitive interviews. Interview protocol for cognitive interviews with patients in phase three.

Interview protocol: cognitive interviews with patients (English*)

*This is a translation from Swedish, provided for publication reasons and might not present a validated translation.

Introduction:

Thank you for helping us improve a questionnaire that is developed and designed to evaluate patients' experiences of healthcare. The questionnaire is aimed at all adults who have been in contact with healthcare, either at a hospital or a healthcare centre. The questionnaire aims to highlight to what extent patients experience that they have been listened to, have been able to participate in decision making processes, and how these processes have led to the development of a plan for continued care. I am a doctoral student at Dalarna University, and I am not involved in the care that you receive here.

Different people have been involved in the development of the questions in the questionnaire. My task is to test the questions suitability at a deeper level using interviews to try to understand how the people answering the questionnaire interpret the questions. I am interested in all your thoughts concerning the questions, both large and small. There are no right or wrong answers and all your reflections are of importance to me. Thoughts about for example a question that you think is unclear, difficult to answer, or how you think when you choose among the different response alternatives are all valuable information to me.

I would like to encourage you to think aloud when answering the questions.

Read the question aloud

Think aloud and tell me what you are thinking:

- when you read the question – what are your thoughts?
- When you are answering a question – what are your thoughts regarding the different response alternatives? Is it possible to choose an appropriate alternative?

… think aloud and tell me about your thoughts, irrespective of whether or not you think they are important!

Tell me if you get into difficulties!

If you forget to talk while you are completing the questionnaire, I may remind you to think aloud. If you feel that it is too difficult to think aloud and fill in the questionnaire at the same time, we can instead go through and discuss your thoughts regarding each question after you have completed the questionnaire.

Once you have completed the questionnaire, I will pose a few questions concerning your thoughts on the different questions and alternative answers.

Protocol **Code:**

Question	Comments
Your experience of the care at the unit (ward) XX<br>This questionnaire contains questions about your experiences of the care at unit (ward) XX during your most recent stay here.<br><br>We define care and treatment as anything done for, or together with you at the unit (ward) XX. Investigation, assessment and planning for continued care and treatment are all assumed to be part of care.<br><br>Sometimes the term" staff" is used in the questions. Staff can be assumed to be all the people you have been in contact with during your stay at unit XX and choose to refer to when answering the questionnaire. Make a note of which people you are referring to when answering the questions:<br><br>☐ Occupational therapist<br>☐ Physiotherapist<br>☐ Doctor<br>☐ Psychologist<br>☐ Therapist<br>☐ Nurse<br>☐ Assistant nurse<br>Other:…………………………………………………………………<br><br>If another person is helping you to answer the questionnaire it is important that they do not influence your answers.<br>Each question is answered using a five graded scale where 1 indicates" No, not at all" and 5" Yes, completely". You can also choose the alternative" Not applicable" if the question has no relevance for you.<br>Answer each question by marking a cross in the box for the answer that best suits your experience.	
1. Did the staff listen to how you experience your state of health/ your illness?	
2. Did you and the staff discuss how your state of health/ your illness can affect your day to day life?	
3. Were you encouraged to ask the questions you wanted to?	
4. If you asked the staff some questions, did you get answers that you could understand?	
5. Did you receive enough information about your care and treatment?	
6. Did you and the staff come to an agreement about the next step in your care and treatment?	
7. Did you participate, to the extent you wished, in decision making about your care and treatment?	
8. Were the things that are important to you in your daily life taken into consideration in the planning of your care and treatment?	
9. Did you and the staff create goals that are important for you in the planning of your continued care and treatment?	
10. Did the staff coordinate your interactions with other health care units to the extent you wished?	
11. Did you and the staff discuss what you yourself can do to improve your state of health/ your illness?	

12. Were your resources, like your will, drive, knowledge, and physical capacity, utilised with regard to your care and treatment?	
13. Were your relatives given the opportunity to participate in your care and treatment to the extent you wished?	
14. Did you have the opportunity to talk with staff if you felt concerned or anxious about your state of health/ your illness or your care and treatment?	
15. Did you feel like an equal person in your meetings with the staff?	
16. Were you met in a way that gave you confidence in the staff?	
17. Did the staff respect you as a person?	
18. Did you and the staff together create a written plan for your continued care and treatment?	
If you have answered YES to question 18 please answer the following two questions. Otherwise continue directly on to the final questions.	
19. Do you feel that you participated, to the extent you wished, in the creation of the plan for your car and treatment?	
20. Was the plan for your continued care and treatment written in a fashion that was understandable to you?	
Free text item. Is there anything more you would like to tell us about your healthcare experience?	

**Examples of prompts:** Regarding questions where patients seem to have had a lot of thoughts, hesitated when answering, chose the highest of lowest answer alternative, thought aloud by saying for example ”this is difficult” and not explained further, had difficulty thinking aloud whilst completing the questionnaire, etc. Use prompts such as ”tell me more”, ”can you give an example”, etc, throughout the think aloud interview. Use prompts to validate what patients are saying if you think it may be unclear such as “ do you mean that…(fill in)” have I understood you correctly…(fill in)”.
How certain are you about your answer?
How difficult was it to answer this question?
Were there any specific questions that you found especially difficult to answer?
What were your thoughts when you chose your answer to this question?
What do you think this question is about?
Do you think it is important to include this question in the questionnaire?
Can you think of any other question that is not at present part of the questionnaire, but which you think should be included?
What is your overall impression of the questionnaire?
