# Supplementary material for: Development and evaluation of the measurement properties of a generic questionnaire measuring patient perceptions of person-centred care
Source: BMC Health Serv Res. 2020 Oct 20;20:960. doi: 10.1186/s12913-020-05770-w (PMC7574493; doi:10.1186/s12913-020-05770-w)
Supplement: Supplementary file 3 — Additional file 3. Content analysis key informants. Subcategories and codes generated using an unconstrained matrix with three pre-defined main categories. [file 12913_2020_5770_MOESM3_ESM.pdf]

Additional file 3. Content analysis key informants

Subcategories and codes generated using an unconstrained matrix with three pre-defined main categories

Main category	Subcategory	Code
Dimensionality (uni- vs. multidimensional concept)	Application of philosophy and an ethical approach in health care contexts	Based in ethics and philosophy
		A premise about human beings translated to actions
	Three interlinked core actions in the same dimension	The three core actions as means for application in practice
		The three interlinked core actions are parts of one dimension
Operationalisation of PCC	Patients perception of core actions and underlying ethical values	Patients' perception of the core actions that reflect the underlying ethical value
		Person centered care more than the three core actions
		Mutual respect
		Dare to give the patient more space
	Listening to patients' narratives	The narrative creates the person
		Listen to take part of patients' knowledge and experience
		Create conditions that enable patients' narrations
	Working in partnership	Creating care *together* with the patients
		Making agreements with the patients
		Build trust
		Identify and use patients' resources
		Information two-way communication
		Provide support
		Common goal setting
	Documentation of agreements	Documentation both in medical record and in documents to the patients
		The patient should understand the documentation
		The patient should recognize herself/himself in the documentation
Patients' perceptions of PCC based on the combined interactions with all health care professionals vs. individual staff members	A patient's experience of care is created by a group of health professionals	Change of culture, environment and atmosphere in the health care
		Common approach as continuity and equality must be ensured
		The underlying ethics and philosophy common for all health professionals
	The group of health professionals is made up of individuals	Development of person-centered approach occur within each individual health professional
		A person-centered approach is more natural for some health professionals
