# Supplementary material for: Development and evaluation of the measurement properties of a generic questionnaire measuring patient perceptions of person-centred care
Source: BMC Health Serv Res. 2020 Oct 20;20:960. doi: 10.1186/s12913-020-05770-w (PMC7574493; doi:10.1186/s12913-020-05770-w)
Supplement: Supplementary file 4 — Additional file 4. Item conceptualisation and I-CVI 1st and 2nd round. Operationalisation of items intended to present PCC in line with GPCC’s concept and I-CVI calculations for first and second Delphi round. [file 12913_2020_5770_MOESM4_ESM.pdf]

Additional file 4. Item conceptualisation and I-CVI 1st and 2nd round. Operationalisation of items intended to present PCC in line with GPCC's concept and I-CVI calculations for first and second Delphi round.

IC	Item	I-CVI 1st round	I-CVI 2nd round
P	1. Have the staff been as much involved in your previous contacts with the healthcare service as you would have liked them to?	0.75	0.88
P	2. Did the staff take into account your own experiences of your illness/health condition?	1.0	1.0
P	3. Did the staff involve you in decisions regarding your care/treatment to the extent that you wished?	1.0	1.0
E	4. Did the staff treat you with compassion and care?	0.88	0.88
P	5. Were you given the opportunity to ask the questions you wanted?	1.0	1.0
P	6. When asking the staff a question, did they reply in a manner that was easy for you to understand?	1.0	1.0
E	7. Did you feel that you were treated with respect and dignity regardless of: Gender, transgender identity or expression, ethnicity, religion or other beliefs, disability, sexual orientation or age?	0.63	0.88
E	8. When asking the staff a question, did they respond with compassionate and sympathetic manner?	0.88	0.75
P	9. Did the care staff involve you in any decisions relating to your healthcare/treatment?	0.88	1.0
P	10. Did the staff involve your relatives in your care and treatment to the extent you wish?	1.0	1.0
P	11. Did you receive sufficient information about your healthcare/treatment?	1.0	1.0
P	12. Did you and the staff come to an agreement on the next step in your rehabilitation/treatment plan?	0.88	1.0
P	13. Did you and the staff discuss how your illness/state of health can affect your daily life?	1.0	1.0
E	14. Were you able to receive emotional support from the staff if necessary (e.g. if you felt worry, fear, anxiety or similar)?	0.88	0.88
E	15. If you were feeling anxious or worried about your condition or treatment, did you have the opportunity to speak to any of the staff about this?	0.88	1.0
D	16. Have you and the care staff created a written care plan for your future care?	1.0	1.0
D	17. If a care plan has been created, did you feel you were involved in its formulation to the extent you would have wished?	1.0	0.88
P	18. Do you think that the staff coordinated your contacts with the health service to the extent that you need?	1.0	1.0
P	19. Did you and the staff discuss what you can do yourself to help improve your health?	1.0	1.0
P	20. Did you feel like the staff treated you as an equal?		1.0
N	21. Did the staff listen to how you experience your illness/health condition?		1.0
D	22. Was your health care plan written in a manner that you understand?		0.88
E	23. Did the staff show consideration to what you told them about your illness/health condition?		1.0
P	24. Did you receive information that you could understand?		0.88
N	25. Were you encouraged by the staff to tell them about your own experience of your illness/health condition?		0.88

(IC) Item conceptualisation, (P) Partnership, (E) Overarching ethical concept, (D) Documentation, (N) Narrative
Item 1–19 were chosen from NP. Item 20–25 are based on suggestions from experts after first Delphi round.
I-CVI calculated for each item as the proportion of experts rating the item as relevant divided by the total number of experts (n=8).
