# Supplementary material for: Development and evaluation of the measurement properties of a generic questionnaire measuring patient perceptions of person-centred care
Source: BMC Health Serv Res. 2020 Oct 20;20:960. doi: 10.1186/s12913-020-05770-w (PMC7574493; doi:10.1186/s12913-020-05770-w)
Supplement: Supplementary file 5 — Additional file 5. Examples of the Delphi process. Examples from the Delphi process, including I-CVI values and comments from experts and resulting revisions. [file 12913_2020_5770_MOESM5_ESM.pdf]

Additional file 5. Examples of the Delphi process. Examples from the Delphi process, including I-CVI values and comments from experts and resulting revisions.

Item from NP	I-CVI and comments 1st round	Revision	I-CVI and comments 2nd round	Revision
5. Were you given the opportunity to ask the questions you wanted?	I-CVI = 1.0<br><br>*Really, I'd rather that the question was read, "Were you encouraged to ask the questions you wanted to ask?"*<br><br>*Change to: Did you feel that there was time for you to ask the questions you wanted to ask?*<br><br>*The patient should have the opportunity not just be given the possibility in my opinion.*	No revision	I-CVI = 1.0<br><br>*Suggested additions…and were you encouraged to ask questions?*<br><br>*Suggested improvements… were you encouraged to ask…*	Were you encouraged to ask the questions you wanted to ask?
7. Did you feel that you were treated with respect and dignity regardless of: Gender, transgender identity or expression, ethnicity, religion or other beliefs, disability, sexual orientation or age?	I-CVI= 0.63<br>*Put an end after dignity. An unnecessary repetition that creates misunderstandings.*<br><br>*Take away: regardless of: Gender, transgender identity or expression, ethnicity, religion or other beliefs, disability, sexual orientation or age?*<br><br>*One needs to think that all patients should be treated like this, regardless of whether the staff members are person-centred or not…*	Did you feel that you were treated with respect and dignity?	I-CVI= 0.88<br>*In my opinion, I would put an end after" respect".*<br><br>*An important question but are the concepts respect and dignity specifically related to PCC?*<br><br>*Look at comments above, can be put together to one question, compassion and respect are enough.*	Did you feel respected as a person by your caregiver/the staff?
16. Have you and the care staff created a written care plan for your future care?	I-CVI = 1.0<br>*Additions: created together*<br><br>*Should it be a health care plan that is written to the patient?*<br><br>*There are many care plans in which the patient is participating but it's not written to the patients in a language they can understand.*<br><br>*It can be called different things –treatment plan, health care plan, plan…*	Did you and the care staff work together to create a written plan for your future care?	I-CVI= 1.0<br>*Staff or caregiver? (your staff sounds a bit odd!)*	Have you and your caregiver/the staff worked together to create a written plan for your future care and treatment?
