# Supplementary material for: Development and evaluation of the measurement properties of a generic questionnaire measuring patient perceptions of person-centred care
Source: BMC Health Serv Res. 2020 Oct 20;20:960. doi: 10.1186/s12913-020-05770-w (PMC7574493; doi:10.1186/s12913-020-05770-w)
Supplement: Supplementary file 6 — Additional file 6. Content analysis cognitive interviews, free-text comments and field notes. Subcategories and codes generated using an unconstrained matrix with four pre-defined main categories. [file 12913_2020_5770_MOESM6_ESM.pdf]

Additional file 6. Content analysis cognitive interviews, free-text comments and field notes. Subcategories and codes generated using an unconstrained matrix with four pre-defined main categories

Main category	Subcategory	Code
Response options and anchors	Unclarity about meaning and interpretation of "written plan" (Q 18, 19, 20)	A verbally agreed but not written health care plan<br>Unclear what a health care plan is
	"Not applicable" used when question does not match patients' situation (Q 13)	No relatives who can participate<br>Do not want relatives to participate
Overall content	The questionnaire captures important aspects of meetings between patients and health care professionals	Feelings of trust towards health care personnel is a prerequisite for high quality care<br>Health care personnel's approach important for high quality care<br>The questions will give a picture of how things are at a health care unit
Responses related to the Rasch analysis	Easy or challenging to choose between response options (response category functioning)	Numbers as response options difficult for some patients<br>Words as response options preferred by some<br>No problems to use numbers as response options
	Overall evaluation of health professionals as a group can be done, but is sometimes challenging (response category function)	Difficult to choose a response option related to all health care professionals in a team or at a health care unit.<br>Experiences of differences in approach amongst health professionals not related to vocational roles<br>Possible to make overall evaluation of health professionals as a group.
	Targeting affected by perceived high levels of PCC at the included units and reluctance to give low ratings (targeting)	Care perceived to be superior at the unit where the patients presently were treated.<br>Responses to questionnaire would be quite different at other units<br>Reluctance to give low ratings on individual items due to high overall satisfaction with the care<br>The 'non-applicable' response option sometimes chosen to avoid low response options
Comprehension and interpretations of the items	Simpler and unambiguous formulations needed	Expression such as 'As much as you wish' and 'enough' are difficult as patients are unaware of what they can get<br>'Resources' unclear term to some patients<br>'Treated as an equal' interpreted in different ways.<br>'Health care plan' both difficult as a term and how to respond if it is not written in collaboration
	Differing views about the relevance and suitability of patients participating in own care and in decisions about own care	Participation in own care and decisions about own care not a task for patients<br>Participation in own care and decisions about own care perceived as highly relevant
